# Alcohol from Coal Gas

**Published:** 1865-01

**Authors:** 


*Alcohol from Coal Gas*.—A method of extracting alcohol from coal gas has been discovered by a young Frenchman, named Cotelle. The cost is stated to be 25 francs the hectolite—about one-third the usual cost.

